# Book Reviews

**Published:** 1903-01

**Authors:** 


### BOOK REVIEWS.

---

Physician's Visiting List (Lindsay & Blakiston) for 1903. Fifty-second year of its publication. Philadelphia, P. Blakiston's Son & Co., 1012 Walnut St. Price $1.00.

This is a very complete and compact memorandum book, bound in flexible black leather. Contains useful information for ready reference, besides blank pages for the physician's use.

---

The Medical News Visiting List for 1903. Weekly (dated, for 30 patients); monthly (undated, for 120 patients per month); perpetual (undated, for 30 patients weekly per year); and perpetual (undated, for 60 patients weekly per year). The first three styles contain 32 pages of data and 160 pages of blanks. The 60-patient perpetual consists of 256 pages of blanks. Each style in one wallet-shaped book, with pocket, pencil and rubber. Seal grain leather, $1.25. Thumb-letter index, 25 cents extra. Lea Brothers & Co., Publishers, Philadelphia and New York.

This contains all the requisites of a visiting list. It is neat, the paper is thin and of excellent quality, and it is readily carried in the pocket without any uncomfortable bulging.

---

Progressive Medicine. Vol. IV. December, 1902. Lea Brothers & Co., New York and Philadelphia.

This volume is concerned with Diseases of the Digestive Tract and Allied Organs, by Max Einhorn; Surgery and Orthopedics, by Joseph C. Bloodgood; Genito-Urinary Diseases, by W. G. Belfield; Physiology, by Albert P. Brubaker; Hygiene, by Charles Harrington; Practical Therapeutics Referendum, by E. Quin Thornton. The volume is uniform with previous issues, and is thoroughly indexed.

